# Hyperoxia arrests pulmonary development in newborn rats via disruption of endothelial tight junctions and downregulation of Cx40

**DOI:** 10.3892/mmr.2014.2192

**Published:** 2014-04-28

**Authors:** CHONG LI, JIANHUA FU, HONGYU LIU, HAIPING YANG, LI YAO, KAI YOU, XINDONG XUE

**Affiliations:** 1 Department of Pediatrics, Shengjing Hospital of China Medical University, Shenyang, Liaoning 110004, P.R. China; 2 Department of Emergency, Shengjing Hospital of China Medical University, Shenyang, Liaoning 110004, P.R. China

**Keywords:** hyperoxia, tight junction, Cx40, permeability, newborn, bronchopulmonary dysplasia

## Abstract

This study investigated changes in vascular endothelial cell tight junction structure and the expression of the gene encoding connexin 40 (*Cx40*) at the early pneumonedema stage of hyperoxia-induced bronchopulmonary dysplasia (BPD) in a newborn rat model. A total of 96 newborn rats were randomly assigned to one of the following two groups, the hyperoxia group (n=48) and the control group (n=48). A hyperoxia-induced BPD model was established for the first group, while rats in the control group were maintained under normoxic conditions. Extravasation of Evans Blue (EB) was measured; the severity of lung injury was assessed; a transmission electron microscope (TEM) was used to examine the vascular endothelial cell tight junction structures, and immunohistochemical assay, western blotting and reverse transcription-polymerase chain reaction (RT-PCR) were used to evaluate the expression of Cx40 at the mRNA and protein level. Our findings showed that injuries due to BPD are progressively intensified during the time-course of exposure to hyperoxic conditions. Pulmonary vascular permeability in the hyperoxia group reached the highest level at day 5, and was significantly higher compared to the control group. TEM observations demonstrated tight junctions between endothelial cells were extremely tight. In the hyperoxia group, no marked changes in the tight junction structure were found at days 1 and 3; paracellular gaps were visible between endothelial cells at days 5 and 7. Immunohistochemical staining revealed that the Cx40 protein is mainly expressed in the vascular endothelial cells of lung tissue. Western blotting and RT-PCR assays showed a gradual decrease in Cx40 expression, depending on the exposure time to hyperoxic conditions. However, the *Cx40* mRNA level reached a trough at 5 days. Overall, our study demonstrated that exposure to hyperoxia damages the tight junction structures between vascular endothelial cells and downregulates Cx40. We therefore conclude that hyperoxia may participate in the regulation of pulmonary vascular endothelial permeability.

## Introduction

Bronchopulmonary dysplasia (BPD) is one of the most serious complications observed in premature infants. In recent years, neonatal mechanical ventilation technology has improved, the antepartum application of hormones and postpartum application of pulmonary surfactants have both become popular, and the survival rate of very low birth-weight infants and extremely low birth-weight infants has gradually increased; however, no change or even, a slight increase in the morbidity associated with BPD have been observed (1,2). A recent survey showed that the incidence rate of BPD in premature infants with a gestational age of <32 weeks is 12–32% (2); however, 97% of pediatric patients with BPD are premature infants with a birth weight of <1,250 g (1). The pathogenesis of BPD is unknown, and no effective therapy is yet available; in addition, the pulmonary development and function of most patients are often affected by long-term exposure to a variety of factors (3,4). Thus, as part of research on neonatal diseases, it is important to explore the pathogenesis and clinical treatment of BPD.

Recent reports have used a hyperoxia-induced model to study BPD; in this model, the early pathological changes of pneumonedema and inflammatory reaction are successfully established (5,6). Factors associated with BPD that have been already extensively studied include reactions to oxidative stress, the release of inflammatory molecules, and epithelial dysfunction (7–10). However, numerous additional factors affect the incidence of pneumonedema, including alveolar epithelial permeability and vascular endothelial permeability. It was previously suggested that in hyperoxia-induced acute lung injury, the pulmonary capillary endothelium is damaged and exposes the capillary basement membrane, thus increasing vascular endothelial permeability (11,12). A preliminary study conducted by our group also reported damage in both the tight junction structures and the function of pulmonary epithelial cells at the early stage of hyperoxia-induced acute lung injury, along with decreased expression of the tight junction protein Zona occludens protein 1 (ZO-1) and of occluding (8). However, there are no relevant reports related to changes in the vascular endothelial tight junction structure.

Connexin (Cx) proteins form intercellular junctions, and these channels allow the movement of small signaling molecules and ions between cells. An association of Cx with the development of numerous lung diseases, such as pulmonary fibrosis, lung injuries and tumors, has been reported (13–16). Previous studies have shown that Cx40 participates in the development of edema and inflammation in certain lung injury processes (15,17,18); however, whether Cx40 affects the progression of hyperoxia-induced acute lung injury is unclear. Therefore, the present study was designed to explore the changes, and their potential effects, in the structure of pulmonary vascular endothelial tight junctions and in the expression of Cx40 at the early stages of hyperoxia-induced lung injury.

## Materials and methods

### Animals and hyperoxic model

Healthy pregnant Wistar rats were purchased from the Center for Experimental Animals of the Shengjing Hospital of the China Medical University (Shenyang, Liaoning, China). All animal procedures were reviewed and approved by the Laboratory Animal Ethics Committee of the China Medical University. The newborn rats were naturally delivered at term and randomly assigned to 2 groups according to their exposure to oxygen: the hyperoxia group was exposed to hyperoxia (90% oxygen) and the control group was exposed to normoxia (21% oxygen) from the day of birth. The establishment of the hyperoxic model is described in our previous paper (8). The inhaled oxygen concentration was measured and continuously recorded using an O_2_ analyzer equipped with a strip-chart recorder (model 572; Servomex Co., Norwood, MA, USA). The room temperature was maintained at 25–27°C, and the humidity routinely set at levels between 50–70%. Nursing rat dams were switched every 24 h between the hyperoxic and normoxic groups to avoid oxygen toxicity, and chambers were open for 10 min per day to add food and clean the cages.

### Sample collection

Solutions containing 2% EB (20 mg/kg body weight) were prepared with physiological saline and injected into newborn rats by cardiac puncture; 30 min later, the newborn rats in each group were initially anesthetized with chloral hydrate and then perfused with heparinized saline via the right ventricle, until a colorless liquid flowed out from the left auricle. At this time-point, the left lung of each animal was separated, the wet weight of the lung after removal of surface water was recorded, and the lung was preserved at −80°C for detection of pulmonary vascular endothelial permeability by EB measurement. Six newborn rats were randomly selected at each time-point (1, 3, 5 and 7 days) and anesthetized with chloral hydrate prior to exposing the thoracic cavity to find the lung and heart. Then, strips of tissue (1×1×2 mm) from the upper right lung of each rat were removed and fixed in 2.5% glutaraldehyde for subsequent observation under a transmission electron microscope (TEM, JEM-1200EX, Hitachi High-Technologies Company, Tokyo, Japan), the left auricles were sheared, and phosphate-buffered saline (PBS) was injected with a right ventricular puncture, until a colorless, clear liquid effused; at this time-point, the complete left lungs were separated and fixed in 4% paraformaldehyde for scoring of lung injury and immunohistochemical assays. The right lung of each rat was separated for Western blotting and reverse transcription polymerase chain reaction (RT-PCR) analyses.

### Lung morphology

Following fixation in 4% paraformaldehyde for 24 h, the lung tissue was dehydrated with graded alcohol, placed in xylene for 1 h and then embedded in paraffin at 60°C. Each section (5 μm) was stained with hematoxylin and examined for histological changes. Twenty areas from each tissue section were randomly selected for exami-nation under a microscope (H600L, Nikon, Tokyo, Japan) at ×400 magnification, and lung injury was scored in each field according to a new histological acute lung injury scoring system (19).

### Detection of pulmonary vascular permeability

Measurements of EB extravasation were performed based on a previously reported method (20). The lung tissue was homogenized in 1 ml of formamide, and the mixture was placed in a water bath and incubated for 24 h at 60°C. The homogenate was then centrifuged at 3,750 g/min for 10 min at room temperature, and the absorbance of the supernatant was measured at 630 nm with an ELx808 spectrophotometer (BioTek Instruments, Inc., Winooski, VT, USA). A standard curve was plotted using the optical density (OD) values of a series of EB dilutions, and pulmonary vascular endothelial permeability was evaluated as the ratio of EB content (μg) in lung tissue to the wet weight (g) of the lung tissue.

### TEM observation of pulmonary vascular endothelial ultrastructures

Lung tissues were fixed in 2.5% glutaraldehyde (Spectrum Chemical, Gardena, CA, USA) overnight at 4°C and washed in PBS (pH 7.4). The tissues were post-fixed in 1% osmium tetroxide (Johnson Matthey Chemicals Ltd., London, UK) and dehydrated in graded ethanol. The tissues were then treated with propylene oxide and embedded in epoxy-resin (Structure Probe Inc., West Chester, PA, USA) embedding medium. The ultrathin tissue sections were stained with uranyl acetate (Resonance Biological Technology Company, Shanghai, China) and lead citrate, and observed using a JEM-1200EX TEM (Hitachi High-Technologies Company, Tokyo, Japan). Images were documented using a SO-163 film (Eastman Kodak Company, Rochester, NY, USA).

### Immunohistochemical detection of the Cx40 protein

Following fixation in 4% paraformaldehyde, the lung tissues were embedded in paraffin and sliced in 4-μm-thick sections. The sections were dewaxed, incubated in 3% H_2_O_2_ for 20 min to eliminate endogenous peroxidase activity, and then incubated in pancreatin (Gibco, Carlsbad, CA, USA) for 20 min. The sections were then washed 3 times with PBS, blocked with rabbit serum, and incubated with a primary antibody targeting Cx40 (Santa Cruz Biotechnology, Inc., Santa Cruz, CA, USA) overnight at 4°C. The tissues were then washed 3 times with PBS, and processed following instructions provided in the UltraSensitive SAP (goat) IHC kit (MX Biotechnology Company, Inc., Fujian, China). Sections were developed using the peroxidase substrate DAB Detection kit (MX Biotechnology Company, Inc., Fujian, China) and were counterstained with hematoxylin. In control experiments, the primary antibody was replaced with PBS. When observed under a light microscope (H600L, Nikon, Tokyo, Japan), cells with brown particles deposited on their membranes were counted as Cx40 positive cells, and images of positive expression areas were recorded.

### Detection of Cx40 in lung tissue by western blotting

Equal amounts of protein (30 μg) were mixed with 1X Laemmli sample buffer (2% SDS, 5% 2-mercaptoehtanol, 10% glycerol, 0.002% bromophenol blue, 0.0625 M Tris HCl, pH 6.8), boiled for 5 min, and electrophoresed on 7.5% precast sodium dodecyl sulfate-polyacrylamide gels (100 V for 120 min). The proteins were then electrophoretically transferred onto polyvinylidene difluoride (PVDF) membranes (40 V for 150 min), and the membranes were incubated for 2 h in Tris buffered saline with Tween-20 (TBST) mixed with skim milk powder (50 g/l), to block nonspecific binding. The PVDF membranes were then incubated overnight at 4°C with primary antibodies targeting Cx40 (1:100) and β-actin (1:10,000, Santa Cruz Biotechnology) diluted in PBS-0.02% Tween-20. Samples incubated in the same solution without primary antibodies served as negative controls. After washing 3 times in TBST, the membranes were incubated with horseradish peroxidase-conjugated secondary antibody (Santa Cruz Biotechnology) at room temperature for 120 min. The membranes were then washed in TBST, developed with the enhanced chemiluminescence (ECL) substrate (Santa Cruz Biotechnology), and the radiographic film was next exposed. Protein bands were visualized with the Chemi Imager 5500 image analysis instrument (Alpha Innotech, San Leandro, CA, USA). Integrated density values (IDVs) were calculated using a computerized image analysis system (Fluor Chen 2.0, Bio-Rad, Hercules, CA, USA) and normalized to the IDV of β-actin.

### Detection of Cx40 mRNA by RT-PCR

Briefly, lung tissue was dissected, homogenized, and RNA was extracted following the manufacturer’s instructions (Takara, Shiga, Japan). RT-PCR was performed with the Prime Script RT-PCR kit (Takara) and the following primers (synthesized by Takara): Cx40 sense, 5′-TCTTTATGATGGCTGTGGC-3′ and antisense, 5′-TGGGCTGTTCTTTAGGC-3′; glyceraldehyde-3-phosphate dehydrogenase (GAPDH) sense, 5′-CGTATCGGACGCCTGGTT-3′ and antisense, 5′-CGTGGGTAGAGTCATACTGGAAC-3′. The amplification reaction was performed in a thermal cycler (ABI, Vernon, CA, USA) under the following cycling conditions: 40 cycles at 95°C for 30 sec, 55°C for 10 sec, and 72°C for 10 sec. PCR products were electrophoresed on 2.5% agarose gels. Gel images were acquired with a Chemi Imager 5500 instrument. The IDVs of PCR products were calculated using the Fluor Chen 2.0, and were expressed relative to the IDV value of the GAPDH gene.

### Statistical analysis

The SPSS 18.0 software (IBM, Armonk, NY, USA) was used for statistical analysis. All data are presented as the mean value ± SD, and the Student’s t-test was employed to analyze inter-group differences between groups at the same time-points, while one-factor analysis of variance and Bonferroni tests were used to test the significance of intra-group differences. P<0.05 were considered to indicate statistically significant differences

## Results

### Lung morphology

**Figure 1:**
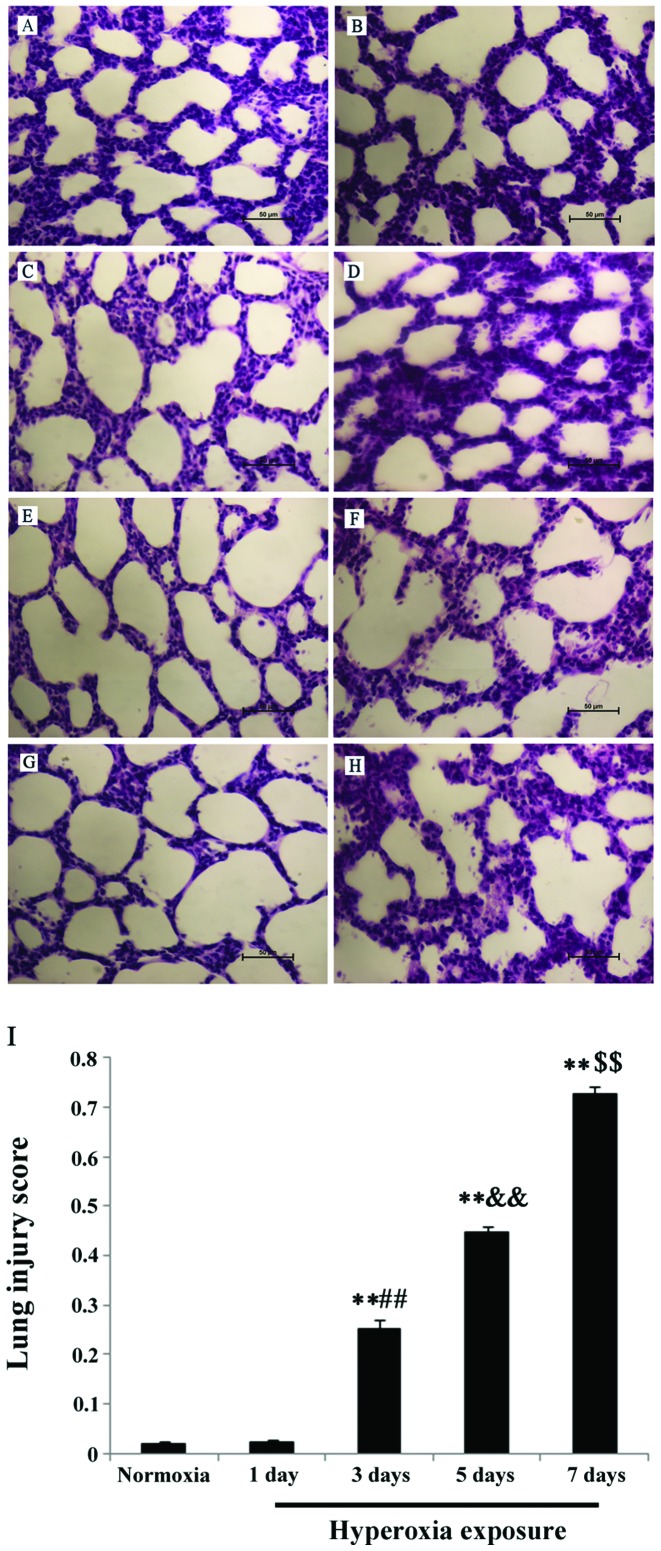
Changes in lung injury at different time-points of exposure to hyperoxia. (A) Examination of the lung tissue morphology in healthy term newborn rats at day 1. (B) After 1 day of hyperoxic exposure, lung tissue morphology does not differ from that of the control group. (C) After 3 days of hyperoxic exposure, thinned pulmonary septa and an increased number of pulmonary alveoli are observed in the control group. (D) At day 3 of hyperoxic exposure, thickened pulmonary septa, vascular engorgement, and neutrophil infiltration into the interstitium are observed. (E) At day 5, the number of pulmonary alveoli is further increased and pulmonary septa are becoming even thinner in the control group. (F) At day 5 of hyperoxic exposure, aggravated pneumonedema, increased neutrophil infiltration in the interstitium, and infiltration of a few inflammatory cells into the alveolar spaces are observed. (G) At day 7, the pulmonary alveolar development of healthy newborn rats demonstrates a uniform and consistent distribution. (H) At day 7 of hyperoxic exposure, pneumonedema is intensified and infiltration of more inflammatory cells is observed, as compared to days 3 and 5. (I) Data from scoring lung tissue morphology, presented as lung injury scores ± SD. ^**^P<0.01 vs. control (normoxia) group; ^##^P<0.01 vs. 1 day of hyperoxia; ^&&^P<0.01 vs. 3 days of hyperoxia; ^$$^P<0.01 vs. 5 days of hyperoxia.

In the control group at day 1, the lung tissues from normal full-term newborn rats showed an irregular alveolus-like structure and a few pulmonary septa (Fig. 1A); at day 3, pulmonary septa became thinner, and an increased number of pulmonary alveoli was observed (Fig. 1C); at day 5, pulmonary septa were even thinner and the number of pulmonary alveoli further increased (Fig. 1E); at day 7, the pulmonary alveoli showed a uniform and consistent distribution (Fig. 1G). In the hyperoxia group, the lung morphology was not significantly different from that of the control group at day 1 (Fig. 1B); at day 3, thickened pulmonary septa, vascular engorgement, and neutrophil infiltration in the interstitium were observed (Fig. 1D); at day 5, evaluation of lung tissues revealed increased infiltration in the interstitium, intensified interstitial edema, and infiltration of inflammatory cells into the alveolar spaces (Fig. 1F); at day 7, a greater degree of lung injury was observed compared to days 3 and 5 (Fig. 1H). These findings showed that lung injury gradually increased over the time-course of exposure to hyperoxic conditions. The scoring results for lung injuries agreed with the results from histological observations, as shown in Fig. 1I.

### Detection of pulmonary vascular permeability

**Figure 2:**
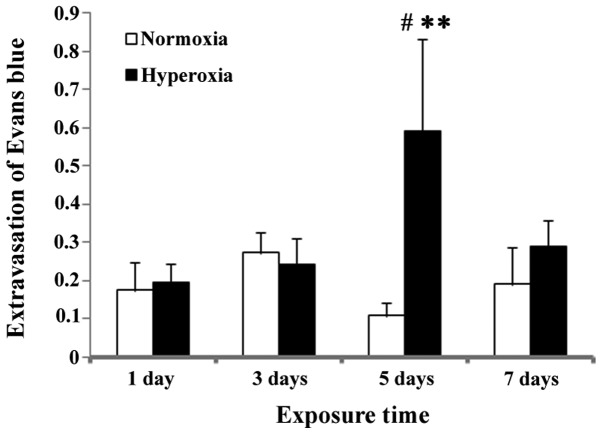
Evaluation of pulmonary vascular permeability in lung tissues. Pulmonary vascular permeability gradually increases upon hyperoxic exposure and reaches a peak at 5 days of exposure. Data are presented as relative EB content (extravasation) ± SD. ^#^P<0.05, compared to other time-points in the hyperoxia group; ^**^P<0.01, compared to the control (normoxia) group at day 5.

From the standard curve obtained using the OD values of different EB concentrations, we established the following formula: Y = 200x + 0.00547, where x denotes EB volume (%) and Y the OD value. We used the above formula to calculate the pulmonary vascular endothelial permeability in the lung tissues, expressed as relative EB content (Fig. 2). The results showed that the relative lung EB content in the hyperoxia group at days 1 and 3 was not different from that of the control group at the same time-points (P>0.05), while at day 5, the relative EB content was significantly higher in the hyperoxia group compared to the control group (P<0.01). In addition, significant differences were observed in the relative EB content of the hyperoxia group between day 5 and other time-points (P<0.05).

### Observation of the pulmonary vascular endothelial ultrastructure

**Figure 3:**
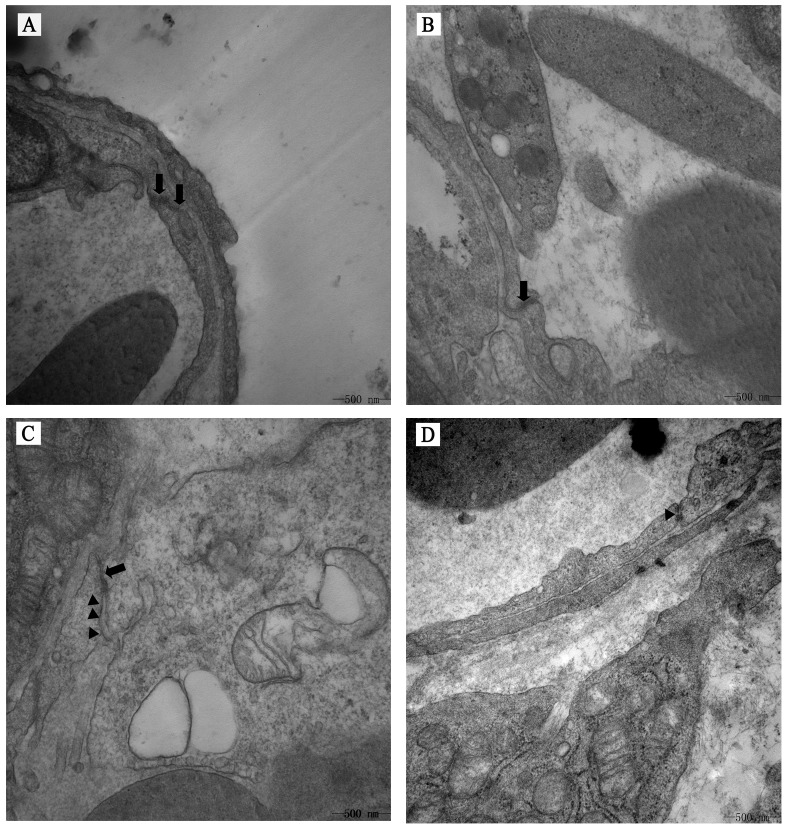
Damage of pulmonary vascular endothelial cell tight junctions caused by hyperoxic exposure. (A) The tight junctions between adjacent endothelial cells were extremely tight in normal newborn rats; (B) At day 1 and day 3 of hyperoxic exposure, no special changes in the tight junction structures were found; (C) At 5 days of hyperoxic exposure, tight junctions were open between adjacent endothelial cells; (D) At 7 days of hyperoxic exposure, tight junctions were intermittent widened.

At all time-points, observations revealed the presence of endothelial cell nuclei with an irregular morphology, intact organelles in the cytoplasm, and intact tight junction structure between endothelial cells, as shown in Fig. 3A. At days 1 and 3 of hyperoxic exposure, no special changes in the tight junction structures were observed (Fig. 3B); at day 5, the tight junctions were open between adjacent endothelial cells (Fig. 3C), and at 7 days, the tight junctions were intermittent widened (Fig. 3D).

### Cx40 localization in Wistar rat lung tissue

**Figure 4:**
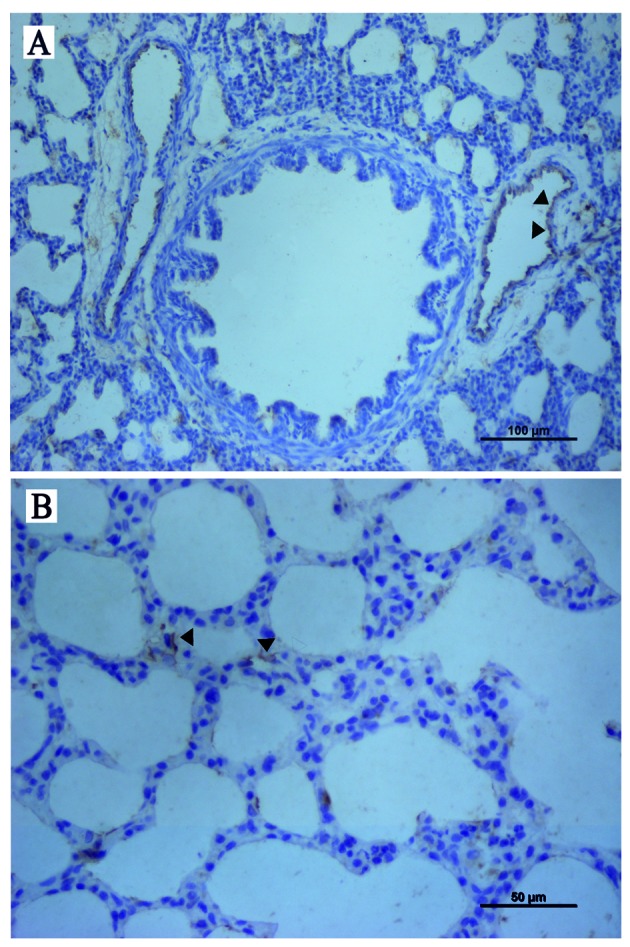
Localization of Cx40 in lung tissues. Positive expression of Cx40 in (A) endothelial cells of larger vessels (hyperoxia day 7, ×200) and (B) capillary endothelial cells of alveolar septa (hyperoxia day 7, ×400).

Light microscopy showed that the Cx40 protein is mainly present in the capillary endothelial cells of alveolar septa and in extra-alveolar vascular endothelial cells, which were stained with brown color, as shown in Fig. 4A and B.

### Cx40 protein detection by western blotting

**Figure 5:**
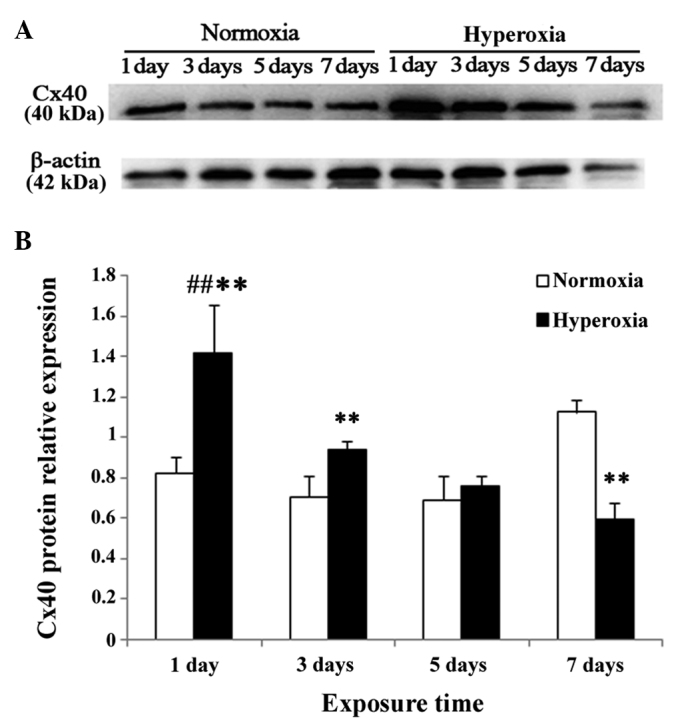
The protein expression of Cx40, assessed by western blotting, shows a gradual decrease along the time-course of hyperoxic exposure. (A) Representative image of western blot and (B) Relative protein level quantification data from the western blots ± SD. ^##^P<0.01 vs. other time-points in the hyperoxia group; ^**^P<0.01 vs. the control (normoxia) group.

The western blotting assay showed that the relative level of expression of the Cx40 protein is markedly increased at day 1 after hyperoxic exposure. Significant differences between the hyperoxia and the control group, and between the 1-day time-point and other time-points, were observed in the hyperoxia group (P<0.01). As the time of exposure to hyperoxia increased, the relative expression level of Cx40 gradually decreased, but remained significantly higher than that of the control group at day 3 (P<0.01); however, it was lower than that of the control group at day 7 (P<0.01), as shown in Fig. 5A and B.

### Cx40 mRNA level in lung tissue

**Figure 6:**
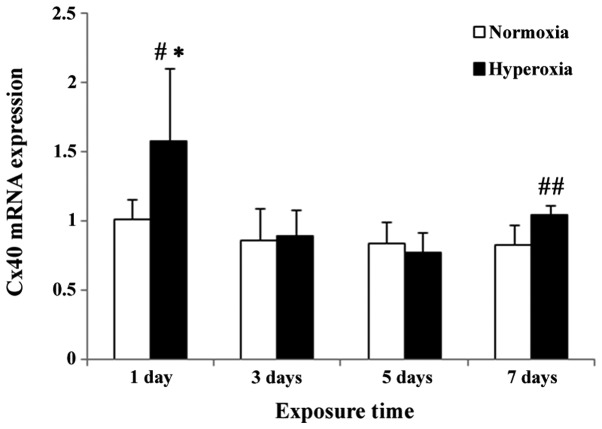
Theexpression of the *Cx40* mRNA, assessed by reverse transcription-polymerase chain reaction (RT-PCR), gradually decreases along the time-course of hyperoxic exposure. Data are presented as relative mRNA level ± SD. ^*^P<0.05, compared to other time-points in the hyperoxia group; ^#^P<0.05, compared to the control (normoxia) group at day 1; ^##^P<0.01, compared to the control group at day 7.

The relative level of the *Cx40* mRNA was markedly increased at day 1 following hyperoxic exposure. Significant differences between the hyperoxia and the control group, and between the relative *Cx40* expression levelsat day 1 and other time-points, were observed in the hyperoxia group (P<0.05). As the time of exposure to hyperoxia increased, the relative *Cx40* mRNA level gradually decreased and reached a trough at day 5 (Fig. 6), but was not statistically different from the corresponding expression level in the control group (P>0.05). However at day 7, the *Cx40* mRNA level in the hyperoxia group was significantly higher than that of the control group (P<0.01).

## Discussion

It was reported that chronic lung injuries induced by exposing newborn animals to hyperoxia are similar to those observed in human BPD in terms of pathology and morphology (21); therefore in this study, a hyperoxia-induced lung injury model was used in rats. Although improvements in perinatal treatment techniques have changed the finally pathological outcome of BPD, the early manifestations remain to be pneumonedema and inflammatory cell infiltration (8,22). Research on the cellular and molecular mechanisms of pathological changes in BPD has focused on the release of inflammatory factors and the role of oxidation and anti-oxidation reactions (6,23,24). Kolliputi *et al* (6) found that the release of inflammatory factors is associated with pulmonary alveolar epithelial permeability, while another study reported that oxyradicals can influence sodium ion transport in a number of distinct pneumonedema models (25). However, therapies that could be potentially used to address the above factors are not yet sufficiently developed for clinical use (26,27). As we observed in a preliminary study, the expression and activity of Na^+^ channels in pulmonary epithelium are increased at the early pneumonedema stage of hyperoxia-induced lung injury, and this increase may relate to pulmonary effusion and absorbance imbalance (22). In addition, we observed damage in the pulmonary epithelial tight junction structures at the acute pneumonedema stage, and that the amounts of the tight junction protein ZO-1 and occludin progressively decreased over the time-course of hyperoxic exposure. These findings suggest that tight junction proteins may participate in the early development of pneumonedema in BPD (8). However, few studies have investigated the mechanism underlying the early changes in pulmonary vascular endothelial permeability following hyperoxia-induced lung injury.

In this study, we examined the changes in pulmonary vascular endothelial permeability by measuring EB extravasation, and showed that pulmonary vascular endothelial permeability gradually increases along the time-course of exposure to hyperoxic conditions; moreover, vascular endothelial permeability reaches a peak at 5 days, but recovers at 7 days of exposure. Tight junctions are the main structures that maintain the barrier function of cells. However in the case of hyperoxia-induced lung injury, whether a change in the tight junction structure leads to increased permeability, in addition to intensifying edema caused by injury in the pulmonary vascular endothelial cells, has not been reported to date. Therefore, in this study, we undertook microscopical examination of the vascular endothelial cell ultrastructure using TEM. TEM observations showed that as the hyperoxic exposure time increased, swelling of endothelial cells and mitochondria occurred, and the number of phagocytic vesicles increased. At 5 days of hyperoxic exposure, the tight junction structure was obviously damaged, and at 7 days, it had become irregular. Based on the above findings, we hypothesize that increased pulmonary vascular endothelial permeability at the early stage of hyperoxia-induced lung injury may relate to the damaged tight junction structure. In a bleomycin-induced lung injury model, Yin *et al* (28) found that the tight junctions of the pulmonary capillary endothelium were an open state at 3 days of treatment with bleomycin, and the number of open tight junctions was significantly higher compared to that of the control group until day 28. In addition, the expression level of the tight junction protein ZO-1 was markedly lower compared to the control group at all time-points following bleomycin treatment. Although a different lung injury model was used in our experiment, we observed damage in the tight junction structures of the pulmonary vascular endothelium at the early stage of hyperoxic exposure, which further supports that tight junctions might play an important role in the development of pneumonedema.

In recent years, the relationship between Cx and the occurrence of lung diseases has become a popular research topic. Studies have suggested that the expression of Cx proteins (Cx40, Cx43, Cx37) is associated with pulmonary fibrosis, lung injury, and lung tumors (29,14,30,16). Cx40 is a gap junction protein widely expressed in pulmonary vascular endothelial cells. In a study of lung injuries produced by gunshot wounds in the chest, Li *et al* (17) observed that Cx40 expression was gradually reduced, pulmonary capillary endothelial permeability was increased, and the immunohistochemical staining intensity of Cx40 correlated with increased capillary permeability. The authors suggested that gap junction channels might affect the change in pulmonary vascular permeability by regulating Ca^+^. However, it is still unclear whether Cx40 participates in the progression of hyperoxia-induced lung injuries. In our study, we detected Cx40 expression at the early stage of hyperoxia-induced lung injury.

In our experiments, we first used an immunohistochemical assay to investigate the localization of Cx40 in Wistar rat lung tissues, and the results showed that Cx40 is mainly expressed in the capillary endothelial cells of alveolar septa and in extra-alveolar vascular endothelial cells. However, it is uncertain whether Cx40-positive cells in the alveolar septa originated from alveolar epithelial cells. Reports related to this question have demonstrated that in mouse lung tissues, Cx40 is only expressed in pulmonary vascular endothelial cells (15,31). We used western blotting to detect changes in the total protein levels of Cx40 in lung tissues at an early stage of hyperoxia-induced lung injury, and the results showed a progressive decrease of Cx40 protein expression along the time-course of hyperoxic exposure. Notably, compared to the control group, the relative protein level of Cx40 showed a markedly increase at day 1 following hyperoxic exposure, and remained significantly lower until day 7. The RT-PCR analysis showed that, changes in the *Cx40* mRNA level at days 1, 3 and 5 of hyperoxic exposure were nearly consistent with those observed at the protein level; at day 7, the relative *Cx40* mRNA level in the hyperoxia group was increased as compared to 5 days, and was significantly higher than the *Cx40* level of the control group. Therefore, we argue that there may be a *Cx40* gene transcription disorder occurring at 7 days of hyperoxic exposure.

Previous studies have shown that in numerous cells, there are 7 Cx proteins (Cx43, Cx45, Cx46, Cx47, Cx50, Cx31.9 and Cx36) connecting with the PDZ region of ZO-1 via their carboxyl terminals (32–34). However, the spatial and functional association of tight junctions and gap junctions in vascular endothelial cells remains unknown. The binding of Cx40 to ZO-1 in vascular endothelial cells has not been reported, but it has been proven that a number of human Cx proteins connect with ZO-1 via their amino acid sequence (32). Therefore, we hypothesize that Cx40 may form a complex with a tight junction by binding to ZO-1, or Cx40 may connect with a tight junction via the Cx40-Cx43 complex (34,35). Nagasawa *et al* (36) found that the functional gap junction and the intercellular signaling factors transmitted in its channel might play a role in maintaining the barrier function of the endothelium, without affecting the expression and subcellular localization of tight junction proteins. Based on the above findings, we conclude that Cx40 may be one of the factors causing increased pulmonary vascular endothelial permeability.

Furthermore, the present study confirmed that the early pathological changes of hyperoxia-induced lung injury consist of inflammatory cell infiltration and pneumonedema, in agreement with previous reports (37,38). Our experiments demonstrated that as the hyperoxic exposure time increased, the degree of injury gradually increased, and at day 7, there were large numbers of inflammatory cells, red blood cells, and amounts of protein exudating in the alveolar spaces. Rignault *et al* (15) used a model of pulmonary inflammatory reaction induced by bacterial lipopolysaccharides, and found that the Cx40 protein level in lung tissue was associated with the number of neutrophils in the bronchoalveolar lavage fluid, and that reduction in Cx40 protein expression was initially observed in the walls of alveoli, i.e., the site where neutrophils migrate. In our experiment, we found that as Cx40 expression decreased, the inflammatory reaction was gradually aggravated; therefore, we infer that reduced Cx40 expression may also relate to the inflammatory reaction.

In summary, our findings show that at the early stage of hyperoxia-induced acute lung injury, the damaged tight junction structure of pulmonary vascular endothelial cells and the decreased expression of Cx40 both play important roles in the development of pneumonedema. However, the specific mechanisms underlying our findings remain to be clarified in a future study, and the results may provide a new target for the early clinical treatment of BPD.
